# Clinical Characteristics and Influencing Factors of Cognitive Impairment in Patients With End-Stage Renal Disease Undergoing Maintenance Haemodialysis

**DOI:** 10.62641/aep.v54i4.2260

**Published:** 2026-08-15

**Authors:** Xiaoxiang Jiang, Xuequn Miao

**Affiliations:** ^1^Department of Nephrology, Cixi People’s Hospital, Wenzhou Medical University, 315300 Cixi, Zhejiang, China

**Keywords:** end-stage renal disease, renal dialysis, cognitive dysfunction, neurobehavioral manifestations, risk factors

## Abstract

**Background:**

This study aimed to investigate the clinical characteristics and influencing factors of cognitive impairment and associated behavioural and psychological symptoms in patients with end-stage renal disease (ESRD) undergoing maintenance haemodialysis.

**Methods:**

This study enrolled 132 patients with ESRD receiving maintenance haemodialysis between January 2021 and January 2025. Patients were divided into a cognitively normal (CN) group and a cognitively impaired (CI) group based on post-dialysis cognitive impairment. The relationships were examined using the Montreal Cognitive Assessment (MoCA) and the Neuropsychiatric Inventory (NPI). Clinical data were collected, and logistic regression analysis was performed to identify factors associated with cognitive impairment.

**Results:**

Compared with the CN group, the CI group had significantly higher scores in abstract thinking, orientation, memory, naming, visuospatial/executive abilities, language fluency, attention and total MoCA scores (*p* < 0.05). On the NPI, the CI group had significantly lower scores (indicating fewer symptoms) than the CN group across all 12 domains, including delusions, hallucinations, agitation/aggression, dysphoria/depression, anxiety, elation/euphoria, apathy/indifference, disinhibition, irritability/lability, aberrant motor activity, nighttime behaviours/sleep disturbances, and appetite/eating disorders, as well as higher total NPI scores (*p* < 0.05). Logistic regression analysis identified age, dialysis duration, and serum levels of haemoglobin (Hb), higher β_2_-microglobulin, 25-hydroxyvitamin D [25(OH)D], and higher intact parathyroid hormone (iPTH) as independent influencing factors associated with cognitive impairment (*p* < 0.05).

**Conclusions:**

Advanced age, longer dialysis duration, lower Hb, higher β_2_-microglobulin, lower 25(OH)D, and higher iPTH levels were associated with cognitive impairment in patients undergoing maintenance haemodialysis for ESRD. Close monitoring of patients presenting with these risk factors is warranted for early prevention of cognitive decline.

## Introduction

End-stage renal disease (ESRD) represents the final stage of chronic kidney disease, characterized by severe renal failure that renders the kidneys incapable of maintaining normal metabolic homeostasis. Haemodialysis is the predominant renal replacement therapy in clinical practice, effectively removing metabolic waste products, maintaining fluid, electrolyte, and acid–base balance and markedly prolonging patient survival [1]. However, long-term haemodialysis can lead to various complications, among which cognitive impairment is one of the most common in patients on maintenance haemodialysis [2]. According to epidemiological data, the prevalence of cognitive impairment among patients with ESRD undergoing haemodialysis ranges from 6.6% to 51% [3]. Such neuropsychiatric abnormalities not only profoundly impair patients’ personal and social functioning but also reduce treatment adherence, raise hospitalization and mortality rates, and severely affect quality of life and long-term prognosis. This has become a critical clinical issue requiring urgent attention.

Cognitive impairment occurs frequently in patients with ESRD on haemodialysis, primarily manifesting as inattention, memory decline, slowed thinking and reduced executive function. In some patients, symptoms are insidious and easily overlooked in the early stages, potentially progressing as the disease advances and even developing into dementia, thereby increasing the risk of adverse events such as falls and non-adherence to dialysis [4]. Behavioural and psychological symptoms frequently co-occur with cognitive impairment, mainly including anxiety, depression, irritability, insomnia, apathy and impulsivity. These symptoms not only exacerbate patients’ psychological distress but also affect communication with family members and social participation, potentially indirectly aggravating renal injury and creating a vicious cycle [5]. Currently, clinical management of patients with ESRD on haemodialysis primarily focuses on maintaining renal function, ensuring dialysis adequacy, and preventing and managing somatic complications, with insufficient attention paid to cognitive impairment and behavioural and psychological symptoms [6].

Despite growing recognition of cognitive impairment in patients on haemodialysis, several important gaps remain. Firstly, most previous studies have focused on global cognitive function rather than domain-specific impairments, leaving uncertainty about which cognitive domains are most affected [5, 6]. Secondly, simultaneous assessment of multiple behavioural and psychological symptom domains in association with cognitive impairment is rarely undertaken. Thirdly, while various serological and demographic factors have been reported, the independent contribution of each factor after controlling for confounders remains inconsistent across studies. Specifically, the roles of β_2_-microglobulin and 25-hydroxyvitamin D [25(OH)D] in cognitive impairment have shown contradictory results in different reports [7].

Therefore, the present study enrolled 132 patients with ESRD undergoing haemodialysis at our institution, aiming to: (1) compare domain-specific cognitive profiles using the Montreal Cognitive Assessment (MoCA) between patients on haemodialysis with cognitively impaired (CI) and patient on haemodialysis with cognitively normal (CN); (2) evaluate the association between 12 categories of behavioural and psychological symptoms (assessed by Neuropsychiatric Inventory [NPI]) and cognitive impairment; and (3) identify independent influencing factors associated with cognitive impairment through multivariate logistic regression analysis. The goal is to provide a more comprehensive reference for the early prevention of cognitive dysfunction in clinical practice.

## Materials and Methods

### Baseline Data

This retrospective study included 132 patients with ESRD who underwent haemodialysis at our hospital between January 2021 and January 2025 through the hospital’s electronic medical record system. All patients met the diagnostic criteria for chronic renal failure, specifically stage 5 chronic kidney disease, as outlined in the Chinese Guidelines for the Early Evaluation and Management of Chronic Kidney Disease (2022 Edition) [8]. Patients were divided into a CN group (n = 47) and a CI group (n = 85) based on MoCA assessments performed during routine clinical care. Cognitive function was assessed using the MoCA, with a total score <26 defined as cognitive impairment [9].

The inclusion criteria were as follows: (1) diagnosis of chronic renal failure and receipt of maintenance haemodialysis for ≥3 months; (2) age ≥18 years; and (3) availability of complete MoCA and NPI assessment records.

The exclusion criteria were as follows: (1) definite prior history of mental illness or history of drug/alcohol dependence; (2) severe visual or auditory impairment, or language comprehension difficulties that would affect scale assessment; (3) concurrent severe dysfunction of major organs such as the heart, liver, or lungs; (4) history of acute cerebrovascular events, traumatic brain injury, or central nervous system infection within the preceding 3 months; or (5) diagnosis of autoimmune diseases or terminal malignancies that could potentially affect cognitive function.

The Medical Ethics Committee of Cixi People’s Hospital, Wenzhou Medical University (approval No. LP-YJ008) approved a waiver of written informed consent for this retrospective study, as the research involved no more than minimal risk to subjects and used existing medical records. Patient data were de-identified prior to analysis, and all procedures complied with the ethical standards of the Declaration of Helsinki.

### Clinical Data Collection

Clinical data for all study participants were retrieved by researchers from the hospital’s electronic medical record system. Supplementary information was obtained through face-to-face questionnaire surveys. Anxiety and depression were assessed using the Hospital Anxiety and Depression Scale (HADS), which consists of 14 items (7 for anxiety, 7 for depression), each rated 0–3. The total score for each subscale ranges from 0 to 21, with higher scores indicating greater symptom severity. A score ≥8 on either subscale was defined as clinically significant anxiety or depression [10]. The collected general data included age, sex, body mass index (BMI), comorbidities, primary disease, dialysis duration, dialysis frequency, dialysis adequacy (assessed by single-pool Kt/V (urea clearance index), calculated from pre- and post-dialysis blood urea nitrogen levels), and the presence of anxiety and depression. Comorbidities encompassed hypertension, diabetes mellitus, and coronary heart disease, diagnosed according to the International Classification of Diseases, 10th Revision (ICD-10) codes: hypertension (I10–I15), diabetes mellitus (E10–E14), and coronary heart disease (I20–I25). Primary diseases included hypertensive nephropathy, diabetic nephropathy, and chronic glomerulonephritis. Diagnostic criteria for hypertensive nephropathy and diabetic nephropathy were based on the Chinese Guidelines for the Prevention and Treatment of Hypertension (2024 Revision) [11] and the Chinese Guidelines for the Prevention and Treatment of Diabetic Kidney Disease (2021 Edition) [12], respectively. Chronic glomerulonephritis was diagnosed based on renal biopsy or clinical diagnostic criteria (persistent proteinuria and haematuria, with or without renal function impairment, after excluding secondary causes) [13]. Dialysis duration was categorized into two strata: ≤3 years and >3 years. Dialysis frequency was categorized as <3 times/week and ≥3 times/week. Kt/V was analysed as a continuous covariate in the multivariate logistic regression models. Serological indicators included white blood cell count (WBC), red blood cell count (RBC), haemoglobin (Hb), β_2_-microglobulin protein, 25(OH)D and intact parathyroid hormone (iPTH).

The following additional variables were extracted from medical records: dialysis adequacy (single-pool Kt/V), occurrence of intradialytic hypotensive events (defined as systolic blood pressure dro*p* ≥20 mmHg or a nadir <90 mmHg requiring intervention, recorded as yes/no for the past 3 months), serum C-reactive protein (CRP, mg/L, measured by immunoturbidimetry), and current use of sedative or antidepressant medications (yes/no).

Serological indicators, including WBC, RBC, Hb, 25(OH)D, β_2_-microglobulin and iPTH, were tested by the hospital’s laboratory. The specific testing methods and instruments were as follows: WBC, RBC and Hb were measured using an automated haematology analyser (model MAL-60 assembly line, Ningbo Meikang Biotechnology Co., Ltd., Ningbo, Zhejiang, China) with original matched reagents (lot numbers varied across detection batches, all passed internal quality control); β2-microglobulin was detected by enzyme-linked immunosorbent assay (ELISA) on Hitachi Model 800 automatic biochemical analyser (Hitachi High-Technologies Corporation, Tokyo, Japan). 25(OH)D was detected by high performance liquid chromatography-tandem mass spectrometry (LC-MS/MS, model Q Exactive Focus, Thermo Fisher Scientific, Waltham, MA, USA); and iPTH was detected by electrochemiluminescence immunoassay using an Abbott ARCHITECT i2000 analyser (Abbott Laboratories, Abbott Park, IL, USA) with Abbott original matching kits (lot numbers varied across detection batches, all passed internal quality control). All tests were performed in strict accordance with the instruments and reagent instructions, and internal quality control results were within the acceptable range.

All clinical assessments and laboratory tests were performed on a non-dialysis day (mid-week, at least 24 hours after the previous dialysis session) to avoid acute effects of fluid shifts and uremic toxin fluctuations. The MoCA and NPI on the same day, prior to the mid-week dialysis session, by trained research assistants who were blinded to the patients’ clinical and laboratory data. Blood samples for serological indicators (e.g., WBC, RBC, Hb, β_2_-microglobulin, 25(OH)D, and iPTH) were drawn from the arteriovenous fistula before the start of the same mid-week dialysis session, following an overnight fast of at least 8 hours.

### MoCA

Cognitive function was screened using the MoCA. This scale, developed by Nasreddine and colleagues through extensive clinical investigation, is a specific screening tool for mild cognitive impairment (MCI). The present study utilized the Chinese version of the MoCA provided by Dr. Vincent Mok of the University of Hong Kong. The scale assesses seven cognitive domains: visuospatial/executive abilities, naming, attention, language fluency, abstract thinking, delayed recall (memory), and orientation, with a total score of 30 points. For all participants with ≤12 years of education (n = 47, 35.6% of the total sample), 1 point was added to the total score to correct for cultural bias. Higher scores indicate better cognitive function. A score of ≥26 points was considered normal, while a score <26 points indicated cognitive impairment. The administration time was approximately 10 minutes per scale. Previous studies have validated the Chinese version of the MoCA, demonstrating good reliability and feasibility, with a Cronbach’s α coefficient of 0.818 [14]. The correlation coefficients between each item score and the total score ranged from 0.5 to 0.9, and the correlation coefficient with the total score of the Chinese version of the Mini-Mental State Examination was 0.933, confirming its effectiveness as a cognitive screening tool [15]. The delayed recall task (5 points) is embedded within the memory assessment; immediate memory is not scored.

### NPI

Behavioural and psychological symptoms were assessed using the Neuropsychiatric Inventory-Questionnaire Informant Version [16]. This scale was developed based on the simplified NPI, and the Chinese version was translated by the Peking University Institute of Mental Health. It is suitable for the rapid assessment of clinical behavioural and psychological symptoms. In the present study, the scale was completed by the patient’s informant (an individual in contact with the patient ≥3 times per week who could accurately describe the patient’s symptoms) to evaluate the presence or absence of 12 types of neuropsychiatric symptoms in the preceding month: delusions, hallucinations, agitation/aggression, dysphoria/depression, anxiety, elation/euphoria, apathy/indifference, disinhibition, irritability/lability, aberrant motor activity, nighttime behaviours/sleep disturbances, and appetite/eating disorders. For symptoms present within the past month, the severity of the symptom (rated 1–3, with higher scores indicating greater severity) and the associated distress experienced by the caregiver (rated 0–5) were assessed. Symptoms not present were rated as “No” and were not scored. Completion time was approximately 10–15 minutes per scale. The total score (range 0–36) was calculated as the sum of the severity scores for the 12 items, with higher total scores indicating more severe behavioural and psychological symptoms. The Chinese version of the Neuropsychiatric Inventory-Questionnaire has been previously validated, demonstrating good reliability and validity, with a Cronbach’s α coefficient of 0.851, a Guttman split-half coefficient of 0.825, and a test–retest reliability intraclass correlation coefficient of 0.860 for the total score (*p* < 0.01) [17]. This confirms its suitability for assessing behavioural and psychological symptoms in the present study population.

### Statistical Analysis

Statistical analysis was performed using SPSS software (version 26.0; SPSS Inc., Chicago, IL, USA; now IBM Corp.). The Shapiro–Wilk test was used to assess normality of continuous variables (*p* < 0.05 indicating non-normal distribution). Normally distributed data are presented as mean ± standard deviation ($\bar{x}$ ± s), and comparisons between two groups were performed using the independent samples *t*-test. Non-normally distributed data are presented as median (interquartile range) (M [P_25_, P_75_]), and comparisons were performed using the Mann–Whitney U test. Categorical variables are presented as frequencies and percentages [n (%)], and comparisons between groups were performed using the chi-squared (χ^2^) test. Multivariate logistic regression analysis was employed to identify risk factors for cognitive impairment. Multicollinearity among the independent variables was first assessed using variance inflation factors (VIF); all VIF values were below 2.5, indicating no marked multicollinearity. A forward stepwise (likelihood ratio) method was used for variable selection. Education level (≤12 years vs. >12 years) was included as a covariate to adjust for its potential confounding effect on the MoCA score-based classification. A two-sided *p*-value < 0.05 was considered statistically significant.

Post-hoc power analysis was performed using G*Power software (version 3.1; Heinrich-Heine-Universität Düsseldorf, Düsseldorf, Germany). For the primary outcome (cognitive impairment status), with a sample size of 132 (85 CI cases, 47 controls) and an alpha level of 0.05, the logistic regression model achieved 86% power to detect an odds ratio of 1.5 for the key predictors (age and iPTH). The minimum detectable effect size (Cohen’s d) for the difference in continuous predictors between groups was 0.28. The achieved power for the main analyses was ≥80%, confirming adequate statistical power.

## Results

### Comparison of MoCA Scores Between the Two Groups

Compared with the CN group, the CI group had significantly lower scores in abstract thinking, orientation, delayed recall (memory), naming, visuospatial/executive abilities, language fluency, and attention, as well as a significantly lower total MoCA score (all *p* < 0.001, Table 1).

**Table 1.** **Comparison of MoCA scores between the two groups ($\bar{x}$ ± s)**.

Domain	CN group (n = 47)	CI group (n = 85)	t	*p*
Abstract thinking	1.82 ± 0.10	1.29 ± 0.18	18.638	<0.001
Orientation	4.92 ± 0.15	3.85 ± 0.62	11.626	<0.001
Delayed recall	4.74 ± 0.25	2.48 ± 0.47	30.622	<0.001
Naming	2.85 ± 0.16	2.21 ± 0.35	11.855	<0.001
Visuospatial/Executive abilities	5.69 ± 0.17	2.92 ± 0.46	39.752	<0.001
Language fluency	2.80 ± 0.19	1.91 ± 0.26	20.607	<0.001
Attention	5.74 ± 0.11	3.65 ± 0.58	24.422	<0.001
Total score	28.56 ± 0.89	22.31 ± 2.15	19.023	<0.001

Note: MoCA, Montreal Cognitive Assessment; CN, cognitively normal; CI, cognitively impaired.

### Comparison of NPI Scores Between the Two Groups

The CI group had significantly higher total NPI scores than the CN group (11.0 [10.0, 12.0] vs. 5.0 [4.0, 6.0], Z = –7.12, *p* < 0.001), indicating more severe behavioural and psychological symptoms in patients with cognitively impairment. The three domains with the largest between-group differences were apathy/indifference, dysphoria/depression, and nighttime behaviours/sleep disturbances (Table 2).

**Table 2.** **Comparison of NPI scores between the two groups [M (P_25_, P_75_)]**.

Domain	CN group (n = 47)	CI group (n = 85)	Z	*p*
Delusions	0.0 (0.0, 0.5)	1.0 (0.5, 1.0)	–5.21	<0.001
Hallucinations	0.0 (0.0, 0.5)	1.0 (0.5, 1.0)	–5.63	<0.001
Agitation/Aggression	0.0 (0.0, 0.5)	1.0 (1.0, 1.0)	–5.94	<0.001
Dysphoria/Depression	0.5 (0.0, 1.0)	1.0 (1.0, 1.5)	–5.78	<0.001
Anxiety	0.5 (0.0, 1.0)	1.0 (1.0, 1.5)	–5.89	<0.001
Elation/Euphoria	0.0 (0.0, 0.5)	0.5 (0.5, 1.0)	–5.91	<0.001
Apathy/Indifference	0.5 (0.0, 1.0)	1.0 (1.0, 1.5)	–5.80	<0.001
Disinhibition	0.0 (0.0, 0.5)	1.0 (0.5, 1.0)	–5.93	<0.001
Irritability/Lability	0.5 (0.0, 1.0)	1.0 (1.0, 1.5)	–5.82	<0.001
Aberrant motor activity	0.0 (0.0, 0.5)	0.5 (0.5, 1.0)	–5.86	<0.001
Nighttime behaviors/Sleep disturbances	0.5 (0.0, 1.0)	1.0 (1.0, 1.5)	–5.88	<0.001
Appetite/Eating disorders	0.0 (0.0, 0.5)	1.0 (0.5, 1.0)	–5.80	<0.001
Total score (0–36)	5.0 (4.0, 6.0)	11.0 (10.0, 12.0)	–7.12	<0.001

Note: NPI, Neuropsychiatric Inventory; CN, cognitively normal; CI, cognitively impaired. Data are presented as median (interquartile range) [M (P_25_, P_75_)]. All comparisons were performed using the Mann–Whitney U test.

### Association Between Specific Behavioural and Psychological Symptoms Domains and Cognitive Impairment

To identify which behavioural and psychological symptoms domains were most strongly associated with cognitive impairment, we performed separate logistic regression analyses with each of the 12 NPI domain scores as independent variables and cognitive impairment status as the dependent variable, adjusting for age and sex (Table 3).

**Table 3.** **Logistic regression analysis of 12 NPI domains associated with cognitive impairment (adjusted for age and sex)**.

NPI domain	OR	95% CI	*p*
Delusions	2.15	1.12–4.13	0.021
Hallucinations	1.98	0.95–4.12	0.068
Agitation/Aggression	2.56	1.32–4.96	0.005
Dysphoria/Depression	3.85	2.01–7.36	<0.001
Anxiety	2.94	1.52–5.68	0.001
Elation/Euphoria	1.52	0.78–2.96	0.221
Apathy/Indifference	4.21	2.18–8.13	<0.001
Disinhibition	2.23	1.10–4.52	0.026
Irritability/Lability	2.78	1.43–5.40	0.003
Aberrant motor activity	1.86	0.92–3.76	0.084
Nighttime behaviors/Sleep disturbances	3.42	1.79–6.54	<0.001
Appetite/Eating disorders	2.45	1.25–4.80	0.009

Note: NPI, Neuropsychiatric Inventory; OR, odds ratio; CI, confidence interval.

The domains with the strongest associations (OR > 3.0) were: apathy/indifference (OR = 4.21, 95% CI: 2.18–8.13, *p* < 0.001), dysphoria/depression (OR = 3.85, 95% CI: 2.01–7.36, *p* < 0.001), and nighttime behaviours/sleep disturbances (OR = 3.42, 95% CI: 1.79–6.54, *p* < 0.001). The remaining domains had ORs ranging from 1.52 to 2.94, with elation/euphoria (*p* = 0.221) and hallucinations (*p* = 0.068) not reaching statistical significance.

### Analysis of Clinical Data of the Two Groups

Among the 132 patients, 47 (35.6%) had ≤12 years of education and received the +1 point correction on the MoCA. After applying the education correction, in the CN group (n = 47), 19 patients (40.4%) had ≤12 years of education; in the CI group (n = 85), 28 patients (32.9%) had ≤12 years of education. The difference in education level distribution between the two groups was not statistically significant (χ^2^ = 0.739, *p* = 0.390, Table 4).

**Table 4.** **Analysis of clinical data of two groups [cases (%), ($\bar{x}$ ± s)]**.

Domain		CN group (n = 47)	CI group (n = 85)	t/χ^2^	*p*
Age (years)		62.38 ± 7.64	68.58 ± 8.74	4.076	<0.001
Education level				0.739	0.390
	≤12 years	19 (40.43)	28 (32.94)		
	>12 years	28 (59.57)	57 (67.06)		
Gender				1.134	0.287
	Woman	22 (46.81)	48 (56.47)		
	Man	25 (53.19)	37 (43.53)		
BMI (kg/m^2^)		23.39 ± 1.35	22.95 ± 1.42	1.734	0.085
Complication					
	Hypertension	21 (44.68)	50 (58.82)	2.435	0.119
	Diabetes	15 (31.91)	38 (44.71)	2.061	0.151
	Coronary heart disease	22 (46.81)	47 (55.29)	0.874	0.350
Primary disease				5.201	0.074
	Hypertensive nephropathy	27 (57.45)	34 (40.00)		
	Diabetic nephropathy	10 (21.28)	34 (40.00)		
	Chronic glomerulonephritis	10 (21.28)	17 (20.00)		
Dialysis time (years)				4.432	0.035
	≤3	30 (63.83)	38 (44.71)		
	>3	17 (36.17)	47 (55.29)		
Dialysis frequency (times/week)				0.113	0.736
	<3	23 (48.94)	39 (45.88)		
	≥3	24 (51.06)	46 (54.12)		
Anxious		9 (19.15)	32 (37.65)	4.836	0.028
Depressed		10 (21.28)	37 (43.53)	6.537	0.011
WBC (×10^9^/L)		6.80 ± 0.53	6.96 ± 0.82	1.205	0.231
RBC (×10^12^/L)		4.49 ± 0.94	4.41 ± 0.85	0.499	0.619
Hb (g/L)		107.45 ± 12.78	92.47 ± 9.41	7.685	<0.001
β_2_-microglobulin (mg/L)		28.45 ± 7.62	52.68 ± 11.35	10.944	<0.001
25(OH)D (ng/mL)		31.17 ± 10.25	20.12 ± 7.09	7.284	<0.001
iPTH (ng/L)		315.79 ± 20.28	532.01 ± 34.22	39.602	<0.001

Note: Data are presented as n (%) or mean ± standard deviation ($\bar{x}$ ± s). CN, cognitively normal; CI, cognitively impaired; BMI, body mass index; WBC, white blood cell count; RBC, red blood cell count; Hb, haemoglobin; 25(OH)D, 25-hydroxyvitamin D; iPTH, intact parathyroid hormone. *p* values were calculated using independent samples *t*-test, Mann–Whitney U test, or chi-squared test as appropriate.

In univariate analysis, older age, longer dialysis duration (>3 years), presence of anxiety and depression, lower Hb, lower β_2_-microglobulin, lower 25(OH)D, and higher iPTH levels were associated with cognitive impairment (all *p* < 0.05). Specifically, compared with the CN group, the CI group had significantly older age (68.58 ± 8.74 years vs. 62.38 ± 7.64 years, t = 4.076, *p* < 0.001), a higher proportion of patients with dialysis duration >3 years (47 [55.29%] vs. 17 [36.17%], χ^2^ = 4.432, *p* = 0.035), higher prevalence of anxiety (32 [37.65%] vs. 9 [19.15%], χ^2^ = 4.836, *p* = 0.028) and depression (37 [43.53%] vs. 10 [21.28%], χ^2^ = 6.537, *p* = 0.011), lower Hb (92.47 ± 9.41 g/L vs. 107.45 ± 12.78 g/L, t = 7.685, *p* < 0.001), lower 25(OH)D (20.12 ± 7.09 ng/mL vs. 31.17 ± 10.25 ng/mL, t = 7.284, *p* < 0.001), and higher iPTH (532.01 ± 34.22 ng/L vs. 315.79 ± 20.28 ng/L, t = 39.602, *p* < 0.001), and higher serum β_2_‑microglobulin levels (52.68 ± 11.35 mg/L vs. 28.45 ± 7.62 mg/L, t = 10.944, *p* < 0.001, Table 4).

### Multivariate Logistic Regression Analysis

With the occurrence of cognitive impairment as the dependent variable (CI vs. CN), and the variables with *p* < 0.05 in univariate analysis (e.g., age, dialysis duration, anxiety, depression, Hb, β_2_‑microglobulin, 25(OH)D, and iPTH) as well as education level as independent variables, multivariate logistic regression analysis was performed using a forward stepwise (likelihood ratio) method. Since anxiety and depression did not satisfy the entry criteria, these variables were not retained in the final regression model (*p* > 0.05). The final model retained seven independent factors (Table 5).

**Table 5.** **Risk factors of cognitive impairment in haemodialysis patients**.

Index	Variable type	B	SE	Wald	*p*	OR	95% CI
Age	Measured value	0.551	0.183	9.066	0.001	1.735	1.212–2.484
Dialysis time	≤3 years = 0, > 3 years = 1	0.616	0.212	8.443	0.018	1.852	1.221–2.807
Education level	≤12 years = 0, >12 years = 1	–0.218	0.176	1.534	0.215	0.804	0.570–1.135
Hb	Measured value	–0.970	0.355	7.466	0.037	0.379	0.189–0.760
β_2_-microglobulin	Measured value	–0.727	0.241	9.100	<0.001	0.483	0.301–0.775
25(OH)D	Measured value	–0.797	0.265	9.045	0.001	0.451	0.268–0.757
iPTH	Measured value	0.994	0.334	8.857	0.006	2.702	1.404–5.202

Note: Dependent variable: presence of cognitive impairment (CI vs. CN). Independent variables: age (continuous), dialysis time (≤3 years = 0, >3 years = 1), Hb (continuous), β_2_-microglobulin (continuous), 25(OH)D (continuous), iPTH (continuous). SE, standard error; OR, odds ratio; CI, confidence interval; Hb, haemoglobin; 25(OH)D, 25-hydroxyvitamin D; iPTH, intact parathyroid hormone.

## Discussion

The present study enrolled 132 patients with ESRD undergoing maintenance haemodialysis. Patients in the CI group had significantly lower MoCA scores and significantly higher NPI scores than those in the CN group. Univariate and multivariate logistic regression analyses identified age, dialysis duration, and serum levels of Hb, β_2_-microglobulin protein, 25(OH)D, and iPTH as independent influencing factors associated with cognitive impairment. Specifically, advanced age, dialysis duration >3 years, low Hb, low β_2_-microglobulin protein, low 25(OH)D, and high iPTH were all associated with an increased risk of cognitive impairment.

Regarding cognitive domains, the most pronounced reductions were observed in the memory and visuospatial/executive abilities domains. In patients with ESRD undergoing haemodialysis, severe renal failure prevents effective clearance of uremic toxins, leading to their accumulation in the body. These toxins can induce oxidative stress and chronic inflammatory responses in cerebral neurons, disrupting neuronal structure and synaptic connections, thereby impairing cognitive function [18]. Visuospatial/executive abilities are closely associated with the function of the frontal and parietal cortices, while memory function depends on normal physiological activity in the hippocampus. The present study extends these findings by identifying memory and visuospatial/executive abilities as the most severely affected domains, which is consistent with the known vulnerability of the hippocampus and frontal-parietal cortices to uremic toxins [19]. Previous studies have focused on global cognitive function or brain network alterations [20, 21], whereas the present study clarifies that within this haemodialysis cohort, memory and visuospatial/executive abilities are the most severely affected domains, providing specific targets for focused assessments and interventions.

On the NPI, the CI group had significantly lower scores across all 12 domains, with the largest differences for dysphoria/depression and apathy/indifference, the physical discomfort associated with ESRD itself and the stress of dialysis therapy can exacerbate these behavioural and psychological symptoms [23]. A potential explanation is that patients with cognitive impairment often exhibit poor insight into their symptoms, resulting in underreporting by patients and their caregiver. Alternatively, caregivers of patients with severe cognitive impairment may gradually become desensitized to behavioural symptoms. Meanwhile, patients with predominant apathy exhibit to display fewer overt neuropsychiatric disturbances. Anees *et al*. [24] found that cognitive impairment was closely associated with mood disturbances, but used depression-specific scales rather than a comprehensive inventory. Wei *et al*. [25] noted an association between neuropsychiatric symptoms and cerebrovascular pathology, but did not compare symptom frequencies between CI and non-CI groups. The present findings suggest the relationship is complex and potentially bidirectional, highlighting the need for objective behavioural assessments in future studies.

Multivariate logistic regression confirmed that age, dialysis duration >3 years, low Hb, low β_2_‑microglobulin, low 25(OH)D, and high iPTH were independent risk factors for cognitive impairment. Elderly patients have degenerative brain changes, and long-term dialysis increases exposure to uremic toxins and vascular injury. Low Hb causes chronic cerebral hypoxia [26, 27]. The relationship between β_2_‑microglobulin and cognition is complex. Some studies have reported that elevated β_2_‑microglobulin is associated with cerebrovascular disease in patients on haemodialysis [28]. However, other evidence suggests that β_2_‑microglobulin is a systemic pro‑aging factor that can impair cognitive function and neurogenesis [29], and it may induce age‑related cognitive decline through mechanisms involving neuroinflammation and apoptosis [30]. In the present study, higher β_2_ with higher odds of cognitive impairment, which may reflect reduced neuroprotective capacity—a finding requiring further validation. Low 25(OH)D compromises neuroprotection, and elevated iPTH disrupts calcium‑phosphorus metabolism, leading to cerebral vascular damage [31]. Elevated β_2_-microglobulin has been identified as a systemic pro-aging factor that impairs cognitive function and neurogenesis, potentially inducing age-related cognitive decline through neuroinflammation and apoptosis pathways. A study by Cui *et al*. [32] found that serum higher β_2_-microglobulin protein levels were closely associated with MCI in the elderly. Shaffi *et al*. [33] identified low 25(OH)D levels as a significant risk factor for cognitive impairment in patients on haemodialysis. However, previous studies have often explored the relationship between single or few indicators and cognitive impairment in patients on haemodialysis, without establishing a systematic risk factor framework [34]. The present study comprehensively analysed multiple categories of variables, including demographic, clinical treatment, and serological indicators, identifying seven independent influencing factors. This contributes to a more comprehensive risk factor profile for patients with ESRD and cognitive impairment receiving haemodialysis, providing a more systematic reference for clinical risk factor screening.

This study is a single-centre retrospective investigation with a relatively limited sample size. Additionally, the sample size precluded formal mediation or moderation analyses to examine whether behavioural and psychological symptoms mediate or moderate the relationships between serological factors (e.g., 25(OH)D, iPTH) and cognitive impairment. Future multicentre, large-sample prospective studies are warranted to further validate these findings and to explore the development of predictive models for cognitive impairment based on the identified risk factors, thereby providing more precise tools for early clinical warning.

## Conclusions

In this retrospective study of 132 patients with ESRD receiving maintenance haemodialysis, cognitive impairment was associated with lower scores across all MoCA domains, particularly delayed recall and visuospatial/executive abilities. Patients with cognitive impairment had fewer behavioural and psychological symptoms on the NPI. Age, dialysis duration >3 years, lower Hb, lower β_2_-microglobulin, lower 25(OH)D, and higher iPTH were independently associated with cognitive impairment. These factors may help clinicians identify high risk patients for closer monitoring.

## Availability of Data and Materials

The datasets used and/or analyzed during the current study were available from the corresponding author on reasonable request.
